# Diagnostic Yield of Microscopic Colitis in Open Access Endoscopy Center

**DOI:** 10.4021/gr339e

**Published:** 2011-07-20

**Authors:** Derek Ellingson, Ronald Miick, Faye Chang, Robert Hillard, Abhishek Choudhary, Imran Ashraf, Matthew Bechtold, Alberto Diaz-Arias

**Affiliations:** a Department of Pathology and Anatomical Sciences, University of Missouri-Columbia, One Hospital Drive, Columbia, MO 65212, USA; b Department of Internal Medicine-Gastroenterology, University of Missouri-Columbia, Five Hospital Drive, Columbia, MO 65212, USA

**Keywords:** Microscopic colitis, Open access endoscopy, Diagnostic yield, Lymphocytic, Collagenous

## Abstract

**Background:**

The diagnostic yield in open access endoscopy has been evaluated which generally support the effectiveness and efficiency of open access endoscopy. With a few exceptions, diagnostic yield studies have not been performed in open access endoscopy for more specific conditions. Therefore, we conducted a study to determine the efficiency of open access endoscopy in the detection of microscopic colitis as compared to traditional referral via a gastroenterologist.

**Methods:**

A retrospective search of the pathology database at the University of Missouri for specimens from a local open access endoscopy center was conducted via SNOMED code using the terms: “microscopic”, “lymphocytic”, “collagenous”, “spirochetosis”, “focal active colitis”, “melanosis coli” and “histopathologic” in the diagnosis line for the time period between January 1, 2004 and May 25, 2006. Specimens and colonoscopy reports were reviewed by a single pathologist.

**Results:**

Of 266 consecutive patients with chronic diarrhea and normal colonoscopies, the number of patients with microscopic disease are as follows: Lymphocytic colitis (n = 12, 4.5%), collagenous colitis (n = 17, 6.4%), focal active colitis (n = 15, 5.6%), and spirochetosis (n = 2, 0.4%).

**Conclusions:**

The diagnostic yield of microscopic colitis in this study of an open access endoscopy center does not differ significantly from that seen in major medical centers. In terms of diagnostic yield, open access endoscopy appears to be as effective in diagnosing microscopic colitis.

## Introduction

Open access endoscopy (OAE) is defined as the performance of endoscopic procedures requested by referring physicians without a prior clinic consultation [1]. OAE is a relatively new development in the field of gastrointestinal medicine and has fallen under close scrutiny for clinical effectiveness. On one hand, bypassing a gastroenterologist consult prior to endoscopy reduces the time patients wait for diagnostic procedures, reduces medical costs, and is reported to have better follow-up care for patients [2, 3]. On the other hand, studies that show disadvantages to open access endoscopy report many patients are inappropriately referred to OAE centers [1, 2]. Also, information vital for patient diagnosis and safety are not properly communicated between the endoscopist and primary-care giver [4]. Additional studies are beneficial to assess the value of OAE.

Studies addressing the diagnostic yield of endoscopy in OAE for all causes are available; among which cancers, adenomas, and inflammatory bowel disease are separately designated [2, 5-7]. At this point, studies to determine the diagnostic yield of biopsies in patients with chronic diarrhea for microscopic colitis in OAE have not been performed.

Microscopic colitis is a treatable illness causing chronic non-bloody diarrhea. Usually, mild or no abnormalities are seen at colonoscopy. The incidence of microscopic colitis varies from 4.2-10% per year per 100 000 people [8]. Biopsies performed for the detection of microscopic colitis yield diagnostic results in 0-5.9% of cases in the United States [9-11]. In studies performed outside the United States, the diagnostic yield in Brazil was 10% and Spain was 9.5% [12, 13]. Other diagnoses established by microscopy, but not always visible by endoscope, include melanosis coli, focal active colitis (also called acute self-limited colitis), and spirochetosis [10].

In this study, we determined the diagnostic yield of biopsies from patients at an open-access endoscopy center in Columbia, Missouri. The information from this study will be useful in the ongoing discussion on the effectiveness of open access endoscopy.

## Materials and Methods

A retrospective study at the University of Missouri-Columbia was performed following approval of the Investigation Review Board. A search of the surgical pathology database was conducted via SNOMED code for specimens, in which the words “microscopic”, “lymphocytic”, “collagenous”, “spirochetosis”, “focal active colitis”, “melanosis coli” and “no histopatholologic” are found in the diagnosis. These keywords represent the scope of diagnoses expected to be given to patients that would have received a colonoscopy for chronic diarrhea with normal colonoscopic findings.

Specimens from 497 consecutive patients at a single private practice, open-access endoscopy center in Columbia, Missouri are obtained and colonoscopy reports are reviewed for the period January 1, 2004 and May 25, 2006. Specimens are accessioned and the tissues and slides are prepared at the University of Missouri. All cases are reviewed and diagnosed by a single pathologist specializing in gastrointestinal pathology.

From this group of specimens, 231 patients were excluded for not meeting the study criteria. Exclusion criteria include patients whose colonoscopy report lists a previous history of inflammatory bowel disease (n = 29), patients with abnormal colonoscopic findings consistent with colitis, such as ulcerations or erythema (n = 35), patients who do not have chronic diarrhea listed as the indication for their colonoscopy (n = 162), and patients whose endoscopy report is missing from our records (n = 5). We chose not to exclude patients with diverticulosis, polyps, or other endoscopic findings unlikely to cause chronic diarrhea.

The diagnosis of lymphocytic colitis is as defined by Lazenby et al [14]. Collagenous colitis was defined as thickening of the sub-epithelial collagen table to greater than 10 mm, with no crypt disruption [15]. Melanosis coli and spirochetosis are also defined in separate studies [16, 17]. Focal active colitis is a term used to describe isolated and nonspecific findings of focal neutrophilic crypt injury [18].

## Results

A total of 266 cases met the clinical criteria for microscopic colitis (i.e. chronic diarrhea and normal colonoscopy). A total of 67 males and 199 females fulfilled the study criteria. The age distribution, mean, and median ages for the total sum of patients and for patients among diagnostic categories are found in Table 1. Of these 266 consecutive patients with chronic diarrhea and normal colonoscopies during the study period, 46/266 (17.2%) patients had a diagnosis with clinical significance. This number includes 12/266 (4.5%) patients with lymphocytic colitis, 17/266 (6.4%) patients with collagenous colitis, 15/266 (5.7%) patients with focal active colitis, and 2/266 (0.8%) patients with intestinal spirochetosis. Besides these diagnoses, 64/266 (24.1%) patients were diagnosed with melanosis coli and 160/266 (60.2%) with no histopathologic abnormality seen. Table 1 Four patients had both melanosis coli and focal active colitis. The diagnostic yield of biopsy for microscopic colitis in our study is 11% (29/266).

**Table 1:** Diagnoses in 266 Patients With Chronic Diarrhea and Normal Endoscopic Findings

Diagnosis	Number Diagnosed	Percentage of Total (%)
Collagenous colitis	17	6.39
Lymphocytic colitis	12	4.51
Spirochetosis	2	0.75
Focal Active Colitis/Ileitis	15	5.64
Melanosis coli	64	24.06

## Discussion

Microscopic colitis is being recognized increasingly as a cause of non-bloody diarrhea in older adults. Our study showed an improved diagnostic yield of microscopic colitis in patients as compared to those published previously worldwide. Given that this difference is an increase rather than a decrease in the diagnostic yield of OAE, there is no evidence to support the supposition that open access colonoscopy is less effective or efficient than colonoscopy performed after specialist referral, as is the practice among many major medical centers and where all previous studies of diagnostic yield in microscopic colitis have occurred.

In some specialty medical centers such as open endoscopy centers, both pathologist and laboratory are employed by and also frequently located in the center. This raises the question of a potential conflict of interest, as endoscopists may have greater incentive to increase their biopsy rates. Because the pathologist reviewing slides and our laboratory are separate from the OAE center, our study is inadequate to assess the influence of this potential conflict of interest that may exist in such a situation.

It is noteworthy that the most recent studies of microscopic colitis have the highest diagnostic yield (Table 2). A significant increase in diagnostic yield exists in this study as compared to studies that have been published previously in the United States [19] and Canada [10], particularly the one performed at this same geographic location previously [9]. This increase in diagnostic yield coincides with a concurrent increase in the incidence of microscopic colitis as noted by population based studies [20, 21].

**Table 2:** Comparison of the Diagnostic Yield Studies for Microscopic Colitis

Authors	Location	Study Period	LC	CC	MC	Total Patients
Prior et al [11]	Manchester, UK	1987	0	2	2	173
Marshall et al [9]	Columbia, MO	1991-1993	0	0	0	111
Lee et al [23]	Korea	1995-1996	1	2	3	118
Patel et al [10]	Manitoba, Canada	1996	-	-	4	173
Fernandez-Banares et al [13]	Spain	1997	-	-	11	115
Shah et al [19]	Cincinnati, OH	1995-1998	10	3	13	218
Da Silva et al [12]	Sao Paulo, Brazil	1996-2000	7	10	17	162
Thijs et al [24]	Netherlands	1999-2000	12	1	13	108
Ellingson et al	Columbia, MO	2005-6	12	17	29	266

CC: Collagenous colitis; LC: Lymphocytic colitis; MC: Microscopic colitis.

Whether this increase reflected a true increase in incidence, an increase in the awareness of the disease by pathologists, or an increase in the number of patients being evaluated by colonoscopy for chronic diarrhea is not known [21, 22]. An increase in diagnostic yield (diagnosis per procedure) over time, as illustrated by the accumulation of studies of diagnostic yield (Table 2), simultaneous to an increase in the incidence (number per capita) over time discredits the latter supposition, since an increase in the number of patients presenting for colonoscopy would not be consistent with a simultaneous increase in both incidence and diagnostic yield. Additional studies would be useful in verifying the trend as well as provide additional clues to determine possible causes of this trend.
